# Pathogenic STX3 variants affecting the retinal and intestinal transcripts cause an early-onset severe retinal dystrophy in microvillus inclusion disease subjects

**DOI:** 10.1007/s00439-021-02284-1

**Published:** 2021-05-11

**Authors:** Andreas R. Janecke, Xiaoqin Liu, Rüdiger Adam, Sumanth Punuru, Arne Viestenz, Valeria Strauß, Martin Laass, Elizabeth Sanchez, Roberto Adachi, Martha P. Schatz, Ujwala S. Saboo, Naveen Mittal, Klaus Rohrschneider, Johanna Escher, Anuradha Ganesh, Sana Al Zuhaibi, Fathiya Al Murshedi, Badr AlSaleem, Majid Alfadhel, Siham Al Sinani, Fowzan S. Alkuraya, Lukas A. Huber, Thomas Müller, Ruth Heidelberger, Roger Janz

**Affiliations:** 1 Department of Pediatrics I, Medical University of Innsbruck, Anichstrasse 35, 6020 Innsbruck, Austria; 2 Division of Human Genetics, Medical University of Innsbruck, Innsbruck, Austria; 3 Department of Neurobiology and Anatomy, MSB 7.046, McGovern Medical School at the University of Texas HSC (UTHealth), 6431 Fannin Street, Houston, TX 77030 USA; 4 University Children’s Hospital, Medical Faculty Mannheim, Heidelberg University, 68167 Mannheim, Germany; 5 Department of Ophthalmology, University Medical Center Halle, Martin-Luther-University Halle-Wittenberg, Halle, Germany; 6 Klinik für Kinder- und Jugendmedizin, Universitätsklinikum Halle, Halle, Germany; 7 Klinik und Poliklinik f. Kinder- u. Jugendmedizin, University of Dresden, Dresden, Germany; 8 Department of Pulmonary Medicine, Division of Internal Medicine, The University of Texas MD Anderson Cancer Center, Houston, TX USA; 9 Department of Ophthalmology, University of Texas Health Science Center, San Antonio, TX USA; 10 Department of Department of Pediatrics, Division of Pediatric Gastroenterology, University of Texas Health Science Center, San Antonio, TX USA; 11 Augenklinik, Universitätsklinikum Heidelberg, Heidelberg, Germany; 12 Erasmus MC-Sophia Children‘s Hospital, Rotterdam, The Netherlands; 13 Department of Ophthalmology, Sultan Qaboos University Hospital, Muscat, Oman; 14 Genetic and Developmental Medicine Clinic, Sultan Qaboos University Hospital, Muscat, Oman; 15 King Fahad Medical City, Children’s Specialized Hospital, Riyadh, Saudi Arabia; 16 Genetics Division and Medical Genomic Research Lab, King Saud Bin Abdulaziz University for Health Sciences (KSAU-HS), Riyadh, Saudi Arabia; 17 Department of Child Health, Sultan Qaboos University Hospital, Muscat, Oman; 18 Department of Genetics, King Faisal Specialist Hospital and Research Center, Riyadh, Saudi Arabia; 19 Division of Cell Biology, Medical University of Innsbruck, Innsbruck, Austria; 20 Present Address: Center for Scientific Review, National Institutes of Health, Bethesda, MD USA

## Abstract

Biallelic *STX3* variants were previously reported in five individuals with the severe congenital enteropathy, microvillus inclusion disease (MVID). Here, we provide a significant extension of the phenotypic spectrum caused by *STX3* variants. We report ten individuals of diverse geographic origin with biallelic *STX3* loss-of-function variants, identified through exome sequencing, single-nucleotide polymorphism array-based homozygosity mapping, and international collaboration. The evaluated individuals all presented with MVID. Eight individuals also displayed early-onset severe retinal dystrophy, i.e., syndromic—intestinal and retinal—disease. These individuals harbored *STX3* variants that affected both the retinal and intestinal *STX3* transcripts, whereas *STX3* variants affected only the intestinal transcript in individuals with solitary MVID. That STX3 is essential for retinal photoreceptor survival was confirmed by the creation of a rod photoreceptor-specific *STX3* knockout mouse model which revealed a time-dependent reduction in the number of rod photoreceptors, thinning of the outer nuclear layer, and the eventual loss of both rod and cone photoreceptors. Together, our results provide a link between *STX3* loss-of-function variants and a human retinal dystrophy. Depending on the genomic site of a human loss-of-function *STX3* variant, it can cause MVID, the novel intestinal-retinal syndrome reported here or, hypothetically, an isolated retinal dystrophy.

**Supplementary Information:**

The online version contains supplementary material available at 10.1007/s00439-021-02284-1.

## Introduction

**Fig. 1:**
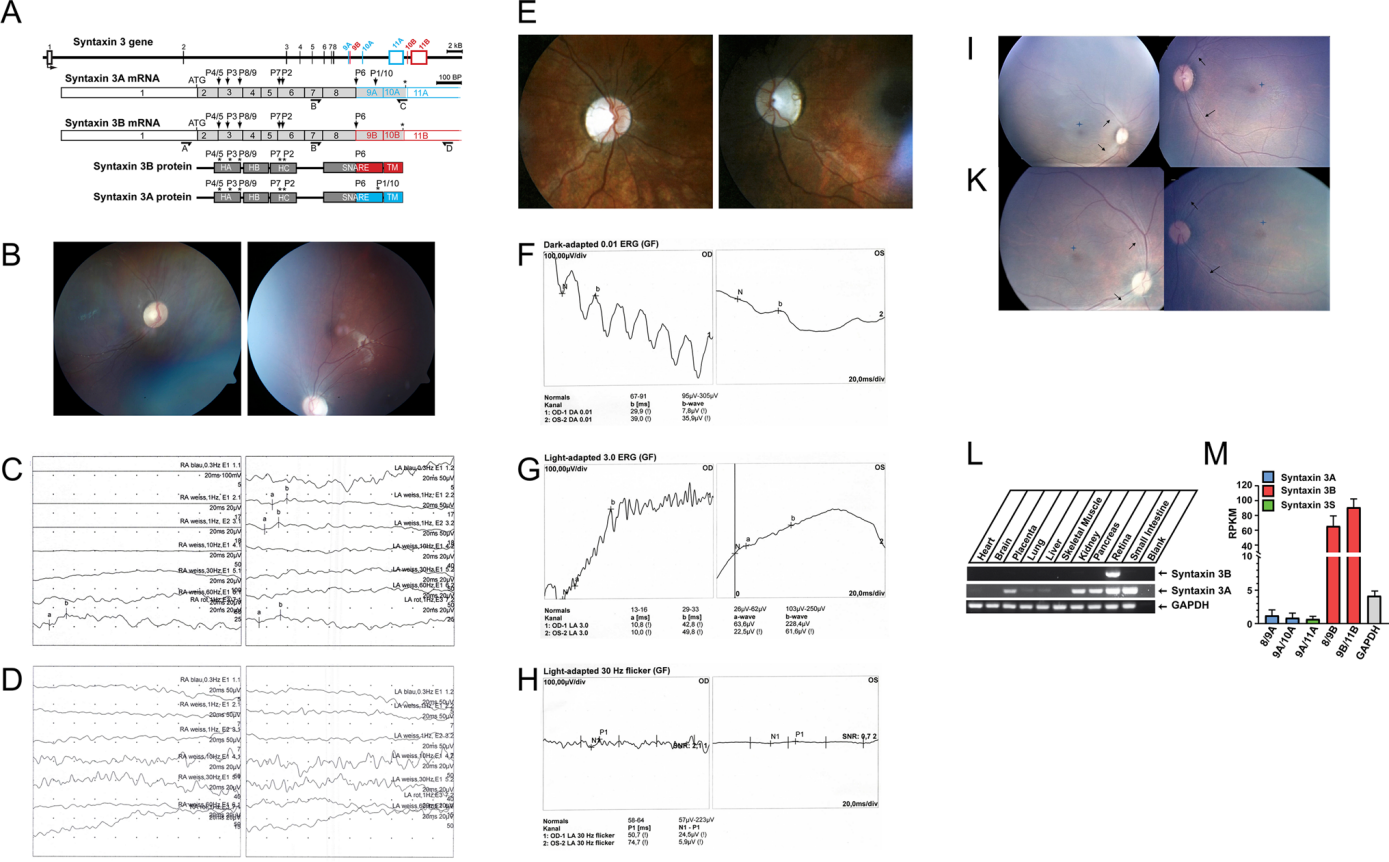
*STX3* structure and human pathogenic variants, the human retinal phenotype, and *STX3* tissue expression. **a** Two major isoforms (STX3A and STX3B) differing in the C-terminal domains are expressed by the STX3 gene; the location of the pathogenic variants in human subjects P1–P10 are indicated. All the pathogenic variants are expected to lead to truncated proteins or nonsense-mediated mRNA decay due to premature stop codons. The pathogenic variant in subjects P1 and P10 only affects the *STX3A* transcript. Primers used for amplification of the human *STX3B* mRNA are indicated. **b** Fundus images of subject P2 at age 10 years. Note the pallor of the optic disk, and mild retinal arteriolar narrowing. Severely reduced amplitudes seen with ERG recordings obtained with skin electrodes in subject P2 at 1 year (**c**) and at 10 years of age (**d**); note, the first 3 ERG traces in (**c**) are flat due to non-recording electrodes. **e** Fundus images of subject P3 at age 5 years. Note the pallor of the optic disk. **f**–**h** Selected ERG traces show a completely flat waveform in subject P3 at 5 years of age. Pallor of the optic disks and attenuated nerve fibers (arrows) in subjects P4 (**i**) and P5 (**k**). **l** STX3B is a retinal-specific transcript in humans. Primers specific for the STX3A and 3B mRNA were used to analyze the expression in different human tissues. GAPDH was amplified as a control. **m** STX3B is the main STX3 isoform expressed in the retina. RNA-sequencing data sets from normal human retina samples were analyzed for the presence of matching reads corresponding to STX3A (exons 8/9A, 9A/10A) and STX3B (exons 8/9B, 9B/10B). Reads per kilobase of transcript, per million mapped reads (RPKM) are shown. (8/9A: 1.2 ± 0.87, 9A/10A:0.83 ± 0.75, 9A/11A: 0.65 ± 0.44, 8/9B: 65 ± 14, 9B/10B:91 ± 12, GAPDH: 4.1 ± 0.74; RPKM ± SD, (*n* = 6))

A severe form of hereditary diarrhea called microvillus inclusion disease (MVID, OMIM 251850) (Vogel et al. 2016) was described in five individuals with biallelic mutations of the syntaxin 3 gene (*STX3*, OMIM 600876) by us and by others (Alsaleem et al. 2017; Julia et al. 2019; Wiegerinck et al. 2014). MVID is characterized by a loss of microvilli, microvillus inclusions, and the accumulation of subapical vesicles in intestinal epithelial cells, suggestive of a trafficking defect (Vogel et al. 2015, 2017). Syntaxin genes code for N-ethylmaleimide-sensitive factor attachment protein receptor (SNARE) proteins that catalyze the fusion between vesicles and their target membranes (Rizo and Sudhof 2012). A major *STX3* spliceform, *STX3A*, is expressed in enterocytes. STX3 is required for the proper trafficking of vesicles to and fusion with the apical membrane in mammalian epithelial cells (Low et al. 1998; Vogel et al. 2015) indicating that the lack of STX3A in epithelial cells causes MVID. We have characterized another transcript, syntaxin 3B, generated by differential splicing and highly expressed in the retinas of mice and fish, where syntaxin 3A mRNA is only expressed at very low levels (Curtis et al. 2008, 2010). Stx3a and Stx3b proteins differ in the C-terminally-located SNARE and transmembrane domains (Fig. 1a). Stx3 localizes to the synaptic boutons of photoreceptors and bipolar cells and to the inner segments of photoreceptors of the rodent retina (Robichaux et al. 2019). It has been implicated in the trafficking of rhodopsin to the outer segments of rod photoreceptors (Chuang et al. 2007; Mazelova et al. 2009), and in the trafficking of peripherin-2 (RDS, PRPH2, OMIM 179605) and rod outer segment protein 1 (ROM1, OMIM 180721) (Zulliger et al. 2015). Finally, a role of STX3 in the release of neurotransmitter at photoreceptor and bipolar cell synaptic terminals was revealed in non-mammalian vertebrate retina (Curtis et al. 2010; Datta et al. 2017; Hays et al. 2020).

We assembled a cohort of ten individuals in eight families, all with MVID, of whom eight displayed a novel syndrome, consisting of MVID and early-onset severe retinal dystrophy (EOSRD). All affected individuals had homozygous loss-of-function *STX3* variants, with a correlation between *STX3* genotypes and intestinal and retinal organ involvement. We demonstrate that *STX3B* is highly expressed in human retina and that the protein is enriched in the inner and outer segments of photoreceptors and in ribbon synapses of the human retina. Finally, we show that the inactivation of *Stx3* in murine rod photoreceptors leads to a progressive degeneration of photoreceptors, corroborating a recently published study that used a different *Stx3* knockout mouse line (Kakakhel et al. 2020). Our study demonstrates that *STX3* is essential for the function of the mammalian retina and that human variants affecting *STX3B* are associated with retinal dysfunction.

## Materials and methods

For all clinical data presented including the molecular genetic studies appropriate informed consent was obtained from subjects or their parents in accordance with the guidelines of the respective institutes at which the subjects were seen and approved by the institutional review boards and ethics committees as required. Human cadaver tissue was donated to the Willed Body Program at the McGovern Medical School at the University of Texas Health Science Center in Houston. Use of cadaver specimens is deemed exempt by the Institutional Review Board.

### Animals

Animal procedures conformed to National Institutes of Health guidelines and were approved by the Animal Welfare Committee of the University of Texas Health Science Center at Houston. Mice were kept under standard housing conditions with unlimited access to food and water and with a 12 h light/dark cycle. Genotyping was performed by PCR using DNA isolated from tail snips as described (Li et al. 2005; Sanchez et al. 2019). Animals did not carry the retinal degenerative mutations RD1 and RD8.

### STX3 variant detection

Novel pathogenic *STX3* mutations were identified in the five new individuals reported here (Table 1). *STX3* mutations were identified by exome sequencing (WES) with DNA extracted from peripheral blood leukocytes in P3, P4 and P5 as described (Klee et al. 2020). For siblings P8 and P9, genome-wide autozygosity mapping analysis was performed with SNP array genotyping (HumanCytoSNP-12v2, Illumina), which highlighted the recently identified MVID gene, *STX3*. Subsequently, the coding exons and flanking intronic sequences corresponding to the STX3A transcript were directly sequenced (primer sequences are available from the authors on request). Variant designations are based on NCBI transcript reference NM_004177.5, using + 1 as the A of the ATG translation initiation codon. The DNA samples from all patients’ parents, and from a number of additional family members were tested for the segregation and zygosity of *STX3* variants identified in index patients (Supplementary Fig. 1).

**Table 1:** Patients’ genotypes and phenotypes

Subject Sex Ethnicity Consanguinity	Reference	*STX3* genotype (homozygous in each case) NCBI references: NM_004177.5, NG_047082.1	STX3 isoforms affected	Ophthalmological findings in subjects with *STX3*-related microvillus inclusion disease (MVID)	Intestinal disease	Additional clinical findings
P1 F White The Netherlands Yes	Wiegerinck et al. (2014)	c.739C > T (exon 9A) p.(Arg247*)	A	Normal vision at 10 years of age	Completely TPN dependent, receiving immunoglobulins once monthly	Severe osteopenia, frequent upper and lower respiratory infections since age 7 years; bronchiectases
P2 M Pakistan Yes	Wiegerinck et al. (2014)	c.372_373dup (exon 6) p.(Arg125Leufs*7)	A/B	Severe visual impairment at 10 years of age, pale optic disk. ERG: progressive bilateral rod/cone dysfunction, first detected at age 1 year, severe at age 10 years	Feeding by gastrostomy tube, small amounts of fluids per os	Mild global developmental delay; failure to thrive (weight − 5.1 z, length − 4.5 z)
P3 F Syria Yes	This publication	c.138del (Exon 3) p.(Ile46Metfs*8)	A/B	Severe visual impairment at 5 years of age. No fixation, nystagmus, pale optic disk VEP: abnormal, ERG: bilateral rod/cone dysfunction at age 5 years	Continuous home PN, partial feeding by gastrostomy tube, small amounts of fluids per os	Motor developmental delay
P4 F Arab Yes	This publication	c.115-2A > G (acceptor splice-site intron 2) skipping of exon 3 r.115_214del p.(Ile39Lysfs*6)	A/B	Severe visual impairment and pale optic disks at 8 years of age. ERG: severe bilateral rod/cone dysfunction	Continuous home TPN	
P5 M Arab Yes	This publication	c.115-2A > G (acceptor splice-site intron 2) skipping of exon 3 r.115_214del p.(Ile39Lysfs*6)	A/B	Severe visual impairment and pale optic disks at 6 months of age		Cousin of P4
P6 F Arab Yes	Maddirevula et al. (2019)	c.675 + 1G > T (donor splice-site intron 8) p.?	A/B	Severe visual impairment at 6 years of age; nystagmus		Global developmental delay
P7 M Afghan Yes	Julia et al. (2019)	c.363_366delinsGA (exon 6) p.(Val122fs*14)	A/B	Severe visual impairment at 20 months of age, congenital nystagmus, and pale optic disks. ERG: severe bilateral rod/cone dysfunction	Completely TPN dependent, gastrostomy tube placed for minimal enteral nutrition	
P8 F Libanon Yes	This publication	c.177_178delCT (exon 3) p.(Tyr60Glnfs*16)	A/B	Severe visual impairment, amaurosis, congenital nystagmus		Died at 10 months of age
P9 M Libanon Yes	This publication	c.177_178delCT (exon 3) p.(Tyr60Glnfs*16)	A/B	Severe visual impairment, amaurosis, congenital nystagmus VEP: abnormal		Sib of P8, died at 5 months of age
P10 M Arab No	Alsaleem et al. (2017)	c.739C > T (exon 9A) p.(Arg247*)	A	None reported; last examined at 8.5 years of age		Mild developmental delay

*TPN* total parenteral nutrition; *PN* parenteral nutrition

We also present clinical follow-up data from five published individuals with *STX3* loss-of-function variants; *STX3* variant identification by WES was reported for P1 and P2 (Wiegerinck et al. 2014) for P6 (Maddirevula et al. 2019), P7 (Julia et al. 2019) and for P10 (Alsaleem et al. 2017). An *STX3* transcript analysis was performed with leukocyte-derived RNA from P4, to assess the effect of a splice-site mutation in that family.

### Tissue preparation for immunohistology

Following euthanasia, mouse retinae were collected, fixed and processed for immunohistochemistry as described (Liu et al. 2014). In brief, eyes were enucleated, the lens and cornea removed, and the resultant retinal eyecups immersion fixed in 4% para-formaldehyde in 0.1M sodium phosphate buffer (pH 7.4) at 4 °C for 24 h. After fixation, eyes were cryoprotected in 30% sucrose/phosphate-buffered saline (PBS, pH 7.4) at 4 °C overnight and then embedded in OCT embedding medium (Tissue-Tek, Torrance, CA), fast-frozen in liquid nitrogen, and sectioned along the vertical meridian on a cryostat at a thickness of 18 µm. These sections were collected on Superfrost Plus microscope slides (Fisherbrand, Pittsburgh, PA) and stored at − 20 °C until use. Human retinal tissue was obtained from three female cadavers (ages 74, 75 and 85). Tissue was collected 4–6 h postmortem. Human eyes were dissected, and a piece of the eyecup was fixed and sectioned as described above for the mouse tissue. For immunolabeling, sections were thawed immediately prior to use, rinsed, and incubated in blocking solution (5% normal goat serum, and 0.3% Triton X-100 in PBS) for 1 h. All antibodies were diluted with blocking solution (5% normal goat serum, and 0.3% Triton X-100 in PBS). Primary antibodies were applied at 4 °C overnight. After rinsing, secondary antibodies were applied for 1 h. Secondary antibodies used at a dilution of 1:300. Double labeling using two primary antibodies raised in different host species was performed by simultaneously applying the primary antibodies and subsequently applying secondary antibodies conjugated to different fluorochromes simultaneously to visualize labeling. Sections were rinsed extensively and cover-slipped in a ProLong Gold antifade mounting medium with DAPI (Invitrogen, Eugene, OR).

### Antibodies

The retinal distribution of STX3 was analyzed with polyclonal antibody (UT478, used at 1:300 dilution) (Liu et al. 2014) and with monoclonal antibody 12E5 (MilliporeSigma, Burlington, MA, United States), which recognize both STX3A and STX3B (Campbell et al. 2020). Monoclonal and polyclonal antibodies against specific markers were used as follows: Mouse monoclonal anti-rhodopsin (used at 1:500–1:1000), Millipore, Co., Temecula, CA (Molday and MacKenzie 1983); rabbit polyclonal anti red/green opsin (used at 1:200–1:500) Millipore Co., Temecula, CA(Otani et al. 2004); mouse monoclonal anti-CtBP2/Ribeye (used at1:200–1:400), Clone 16/CTBP2 BD Biosciences, San Jose, CA (against Mouse CtBP2 aa. 361–445), Syntaxin 3 mouse monoclonal clone 12E5, MilliporeSigma (Burlington, MA, United States) (used at 1:200 dilution).

Secondary goat-anti Ig and goat-anti rabbit mouse Ig conjugated to Cy3 (Jackson ImmunoResearch, West Grove, PA) or Alexa Fluor 488 (Molecular Probes, Eugene, OR) where used for labeling. All antibodies were diluted with blocking solution (5% normal goat serum, and 0.3% Triton X-100 in PBS). Specificity of immunolabeling was confirmed by processing a second set of sections in the absence of the primary antibodies, or by substituting normal rabbit serum for polyclonal antibodies, as appropriate.

### Imaging

Images were captured with a ZEISS LSM 800 confocal microscope (San Diego, CA) or with a Nikon A1R Confocal Laser Microscope (Hercules, CA) at a thickness of 0.3–0.5 µm. Confocal images were processed using the manufacturer’s software Zen 2.5 (Blue edition) or Nikon Imaging Software Element (Version 4.20) and with Adobe Photoshop.

### Transcript expression analysis

The expression of the different *STX3* transcripts was analyzed by identifying matching reads from recent Illumina TruSeq® RNA-seq projects obtained with human retina samples (Ratnapriya et al. 2019). Datasets from retinas from six control donors selected for age (47–57 years) were analyzed using the BLAST program of the Sequence Read Archive database (SRA) at the NCBI (https://blast.ncbi.nlm.nih.gov/Blast.cgi?PROGRAM=blastn&BLAST_PROGRAMS=megaBlast&PAGE_TYPE=BlastSearch&BLAST_SPEC=SRA&SHOW_DEFAULTS=on) (individual specimen numbers: SRS3492913, SRS3493239, SRS3493240, SRS3493261, SRS3493276, SRS3493282). Probes of 120 bp length covering 60 bp upstream and downstream of the respective splice sites were analyzed. Default BLAST settings were used with the exception of the Expect threshold, which was set to 1000. Reads were identified as matching if they had at least 90 bp identity with the probe, corresponding to an *E* value of < 10^–30^. Reads per kilobase of transcript, per Million mapped reads (RPKM) where calculated for each probe.

Sequences of probes used:

HS STX3 8_9B

AGCAGCATCAAGGAGCTTCACGACATGTTTATGGACATCGCCATGCTGGTGGAGAATCAGGGATCCATGATTGACCGTATTGAGAACAACATGGACCAGTCAGTGGGCTTTGTGGAGCGG

HS STX3 9B_10B**:**

GTGGAGCGGGCCGTGGCAGATACCAAAAAGGCTGTCAAGTATCAGAGTGAAGCCCGGAGGAAGAAGATCATGATCATGATCTGCTGTATTATCCTTGCGATCATCTTAGCTTCCACCATT

HS STX3 8_9A**:**

AGCAGCATCAAGGAGCTTCACGACATGTTTATGGACATCGCCATGCTGGTGGAGAATCAGGGTGAGATGTTAGATAACATAGAGTTGAATGTCATGCACACAGTGGACCACGTGGAGAAG

HS STX3 9A-10A**:**

AATAATCAATGCTAAAATGCCCAGCAACACAACTACTAGCACAATGATAATTATCAATTTCTTCCGGGCCTGACTCTGGTATTTCACAGCTTTTTTCGTTTCATCTCGTGCCTTCTCCAC

HS STX3 9A-11A**:**

GTGGAGAAGGCACGAGATGAAACGAAAAAAGCTGTGAAATACCAGAGTCAGGCCCGGAAGAAACTGATTTCACTCCAGACTGGTGTGGCCACCCTTGTCTTCAGATGAGAATGGAGTCTG

### STX3 analysis of human tissues

Human Tissue cDNA samples were purchased from Takara Bio USA, Inc. (Mountain View, CA). Standard PCR reactions were performed with the OneTaq Hot Start Quick kit (New England Biolabs) using these primers:

- A: HS Syntaxin 3AB Sense exon 1:GGCTTCAGGATGAAGGACCG
- B: HS Syntaxin 3B Sense Exon 7:GCAACCCGGCCATCTTCACTTCTG
- C: HS Syntaxin 3A Antisense exon 10A:AGCCCAACGGAAAGTCCAAT
- D: HS Syntaxin 3B Antisense Exon 11B:CCTGTCCCTGTCCTCCGCCCAAT
- GAPDH1: ATGACATCAAGAAGGTGGTG,
- GAPDH2: CATACCAGGAAATGAGCTTG

Conditions: 35 cycles for each reaction, annealing temperatures STX3: 55 °C; GAPDH: 50 °C. PCR reactions were analyzed using agarose gel-electrophoresis and correctness of the amplified fragments was confirmed by direct sequencing.

## Results

### Pathogenic variants in the human STX3 gene that affect STX3B are associated with retinal disease

Given the high level of *STX3* expression in the murine and non-mammalian retina and roles for Stx3 in protein trafficking in photoreceptors (Chuang et al. 2007; Kakakhel et al. 2020; Mazelova et al. 2009; Zulliger et al. 2015), and in neurotransmitter release at the first two synapses in the visual throughput pathway (Curtis et al. 2008, 2010; Datta et al. 2017; Liu et al. 2014) we asked whether MVID subjects with *STX3* variants might have visual impairment. To address this question, we studied five newly identified MVID subjects in which we found homozygous *STX3* variants; in addition, we investigated five previously described MVID subjects with *STX3* variants (Alsaleem et al. 2017; Julia et al. 2019; Maddirevula et al. 2019; Wiegerinck et al. 2014) for the presence or absence of symptoms of marked visual impairment. In all affected individuals, the onset of diarrhea was in the first week of life, there was persisting diarrhea and histopathology demonstrated the characteristic features of MVID (not shown). The genotypes of each subject, along with visual testing results, are summarized in Table 1; Fig. 1a depicts the positions of all seven identified *STX3* variants, with each of the 10 subjects indicated. Supplementary Fig. 1 shows the pedigrees, segregation and chromatograms relating to novel STX3 variants. Importantly, each of these *STX3* variants results in a premature stop codon, expected to either lead to nonsense-mediated mRNA decay or to C-terminally truncated STX3 protein that lacks the transmembrane domain; the latter is thought to be essential for STX3 function (Rizo and Sudhof 2012). Thus, each of the identified human mutations is expected to behave as a loss-of-function mutation. Eight of the ten subjects (P2-P9) had pathogenic variants that were located in exons shared between the *STX3A* and the *STX3B* transcripts, and these eight subjects would be predicted to have loss of *STX3A* and *STX3B* expression. In contrast, two of the subjects (P1, P10) carried the same pathogenic variant (c.739C > T, p.Arg247*) that is located in exon 9A and thus spares STX3B.

Severe visual impairment was seen and a diagnosis of EOSRD rendered in individuals P2-P9, as evidenced by the inability to respond to or track visual stimuli, locate or reach for objects. Half the subjects also exhibited nystagmus. Five subjects underwent fundoscopic examination, revealing pallor of the optic disks (Fig. 1b, e, i, k). Unfortunately, because these subjects are both young and ill due to their MVID, with two sibling subjects (P8 and P9) succumbing to MVID-associated problems at less than one year of age, our ability to assess visual function more rigorously or conduct longitudinal studies was limited. In five subjects, electroretinography (ERG) demonstrated severe rod and cone dysfunction in both eyes. In the case of subject P2, ERG recordings with skin electrodes (Bradshaw et al. 2004) and fundus images were obtained at 1 year and at 10 years of age. The first ERG showed A and B waves that were greatly reduced in amplitude (Fig. 1c), consistent with a dramatic decrease in the light responses of photoreceptors and downstream bipolar cells, respectively. By 10 years of age, the A and B waves were almost entirely absent in this subject (Fig. 1d), suggesting a progressive loss in the light-sensing and signaling ability of the retina. Marked reductions in the A and B waves were also seen in P3 at 5 years of age (Fig. 1f–h). Subject P7, reported at age 15 months with nystagmus and visual impairment (Julia et al. 2019) had flat ERG recordings at 20 months of age indicating severe photoreceptor dysfunction (not shown). Subjects P8 and P9 had abnormal visual evoked potentials (VEP) responses. VEPs evaluate the integrity of the visual pathway from the retina to the visual cortex, but given the paucity of STX3 expression in the brain (see Fig. 1l, m) these VEP abnormalities are most likely of retinal origin. By contrast, subjects P1 and P10 that carried the STX3A-only variant did not have visual impairment upon clinical inspection at 10 and at 8.5 years of age. Together, the ophthalmological findings obtained in MVID subjects point towards an essential role for STX3 in the retina.

### STX3B is a transcript selectively expressed in the human retina

Given the visual deficits in MVID subjects with pathogenic variants in both *STX3A* and *STX3B*, we asked whether both splice forms were present in the human retina. A GeneBank search identified a large number of ESTs and several full-length cDNA clones corresponding to *STX3A*. However, while no clones corresponding to *STX3B* were identified, analysis of the human STX3 gene sequence showed the presence of putative *STX3B*-specific exons 9B, 10B and 11B (Fig. 1a).

We find that the human STX3B transcript is generated by differential splicing in the same pattern as observed in the mouse, and expressed only in the retina (Fig. 1a, l). *STX3A* and *STX3B* share exons 1–8 and differ with respect to exons 9–11. STX3A and STX3B differ in half of their SNARE and in their C-terminal transmembrane domains.

For *STX3A*, we detected strong signals in human small intestine, kidney, pancreas, placenta as well as in retina, with weaker expression levels detected in lung, liver and heart, and no signals above background detected in human brain and skeletal muscle tissues (Fig. 1l). However, the commercial human retina cDNA samples (Takara Bio USA, Inc.) used in our experiments were obtained from pooled retinas from 99 donors and contained not only the neural retina, but also the retinal pigmented epithelial cell layer (RPE) (per manufacturer).

We next compared the relative levels of *STX3A* and *STX3B* mRNA in the human retina by analyzing the presence of splice junctions specific for the two isoforms in RNA-seq data sets from six normal human retina samples (Ratnapriya et al. 2019), and included a soluble form of *STX3*, *STX3S* that is generated by splicing together exons 9A and 11A and skipping exon 10A (Giovannone et al. 2018). These data demonstrate that *STX3B* is the major human retinal transcript (Fig. 1m).

### STX3 protein is located at retinal ribbon synapses and in the outer and inner segments of photoreceptors of the human retina

To better understand the role of STX3 loss-of-function in our human cohort, we examined the expression and subcellular localization of the STX3 protein in the human retina via immunohistochemistry with STX3 antibodies that detect both STX3A and STX3B (Campbell et al. 2020; Liu et al. 2014; Zulliger et al. 2015). Given that our mRNA expression analysis of human retina showed that STX3A corresponds to only about 1% of total STX3 mRNA (Fig. 1m), the observed STX3 signal in human retina most likely corresponds almost exclusively to STX3B, as it does in the mouse retina (Curtis et al. 2008).

**Fig. 2:**
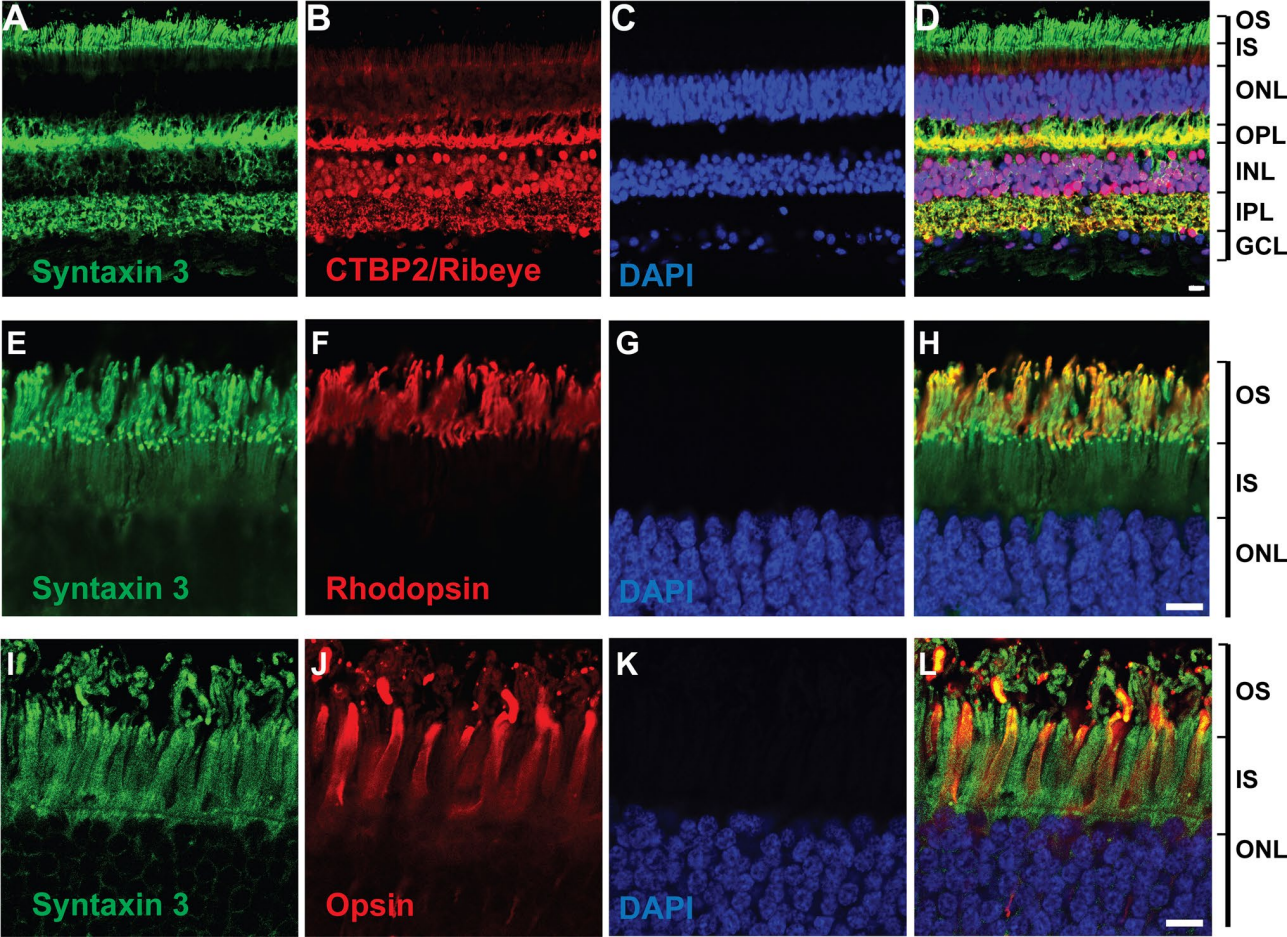
STX3 is found in the synaptic terminals and the inner and outer segments of the photoreceptors and in bipolar cells in the human retina. Human retina was labeled with antibodies against STX3, CTBP2/Ribeye (**a**–**d**), rhodopsin (**e**–**h**), red/green cone opsin (**i**–**l**) and nuclei were counterstained with DAPI (nuclear marker). Retinal layers are labeled on the right (Inner segments IS, outer segments OS, outer nuclear layer ONL, outer plexiform layer OPL, inner nuclear layer INL, inner plexiform layer IPL and ganglion cell layer GC). This is a representative example and similar results were obtained in *n* = 3 donor samples. Scale bars: 20 μm

**Fig. 3:**
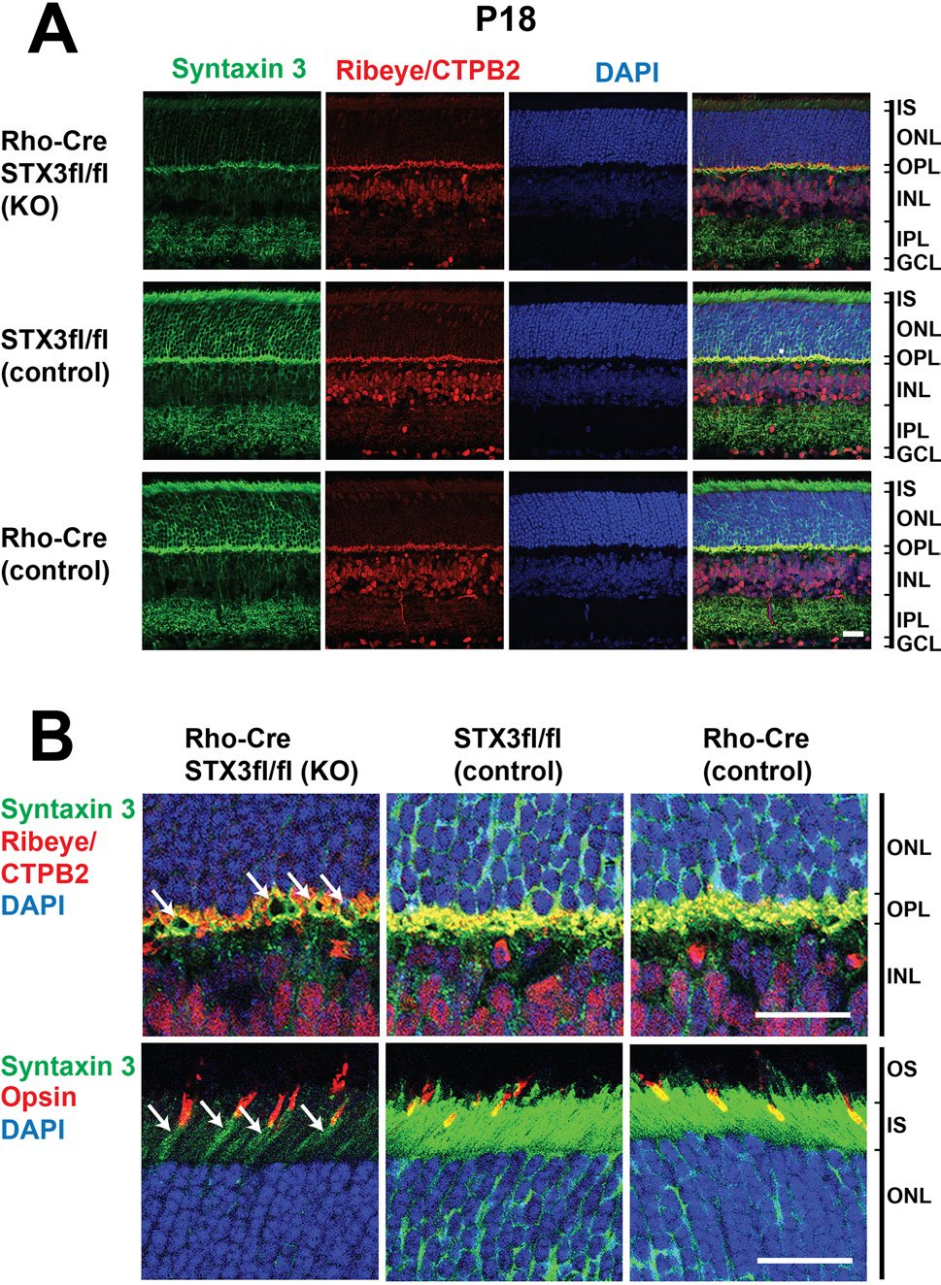
At 18 days of age, conditional Stx3 knockout mice confirm an absence of Stx3 in rods with otherwise normal retinal morphology. **a** Representative vertical sections of retina from conditional Stx3 knockout mice (Rho-Cre/STX3fl/fl) and their controls were labeled with antibodies against Stx3, CtBP2/Ribeye and DAPI. Note normal retinal architecture but a lack of Stx3 immunolabeling of knockout mice in the IS, ONL and OPL. **b** Top panel, higher magnification of the OPL showing lack of Stx3 protein label in the small rod terminals of the conditional knockout mice with Stx3 still present in the larger cone terminals marked by the arrows. Bottom panel. Labeling with antibodies against Stx3, red green opsin (cone marker) and DAPI confirms that the remaining Stx3 expressing cells are cone photoreceptors. The arrows point to the remaining Stx3 signal found in the inner segments of cones. *IS* Inner segments, *OS* outer segments, *ONL* outer nuclear layer, *OPL* outer plexiform layer, *INL* inner nuclear layer, *IPL* inner plexiform layer, *GCL* ganglion cell layer. Scale bars: 20 μm

Photoreceptor terminals and bipolar cell terminals, located in the outer plexiform layer (OPL) and inner plexiform layer (IPL) respectively, labeled for the major structural protein of the synaptic ribbon, ribeye, exhibited strong STX3 immunolabeling (Fig. 2a–d), consistent with a role for STX3 in neurotransmitter release and the conveyance of visual information to downstream neurons in the human visual pathway. Ribeye shares a domain with the nuclear protein CtBP2, and thus CtBP2 antibodies label synaptic ribbons in addition to some nuclei, such as those in the inner nuclear layer (INL). Strong STX3 immunolabeling of photoreceptor inner and rhodopsin-labeled outer segments was observed in the human retina (Fig. 2e–h), contrasting with the labeling of only inner segments in mouse photoreceptors (Fig. 3) (Chuang et al. 2007; Zulliger et al. 2015). In addition, the inner and outer segments of cone photoreceptors also exhibited immunolabeling for STX3 in the human retina (Fig. 2i–l). The labeling of the plexiform layers and both inner and outer segments was observed with both STX3 antibodies and in retinal sections obtained from three different donors.

### Inactivation of the Stx3 gene in mouse rod photoreceptors leads to degeneration

To probe for an essential role of Stx3 in vision, we generated a cell-specific Stx3 mouse knockout line, as complete inactivation of the Stx3 gene in mice produces an embryonic lethal phenotype (Sanchez et al. 2019). A rod photoreceptor-specific inactivation of *Stx3* was achieved with a mouse line that expresses an optimized iCre under the control of the rhodopsin promoter (Li et al. 2005; Sanchez et al. 2019). Cre recombinase activity is first detectable in the retina of the Rho-iCre mice at 7 days of age, and it reaches a plateau at 18 days (Li et al. 2005). Our breeding scheme generated the conditional *Stx3* knockout mice (fl/fl; Rho-cre/ +), as well as homozygous *Stx3* floxed control mice (fl/fl; + / +) and heterozygous Cre expressing control mice (+ / + ; Rho-cre/ +).

Whereas the retinal architecture, cell morphology, and number of cells in the nuclear layers appeared grossly normal at day 18, there was a reduction in Stx3 immunolabeling in the OPL and in the inner segments of the photoreceptor layer of the fl/fl; Rho-cre/ + mice relative to controls (i.e. fl/fl and Rho-Cre). Double-labeling for Stx3 and ribeye/CtBP2 and for Stx3 and cone opsin revealed that the remaining Stx3 signal was in cone photoreceptors (Fig. 3b). Stx3 immunoreactivity appeared normal in the IPL, where the synaptic terminals of the bipolar cells reside (Fig. 3a).

**Fig. 4:**
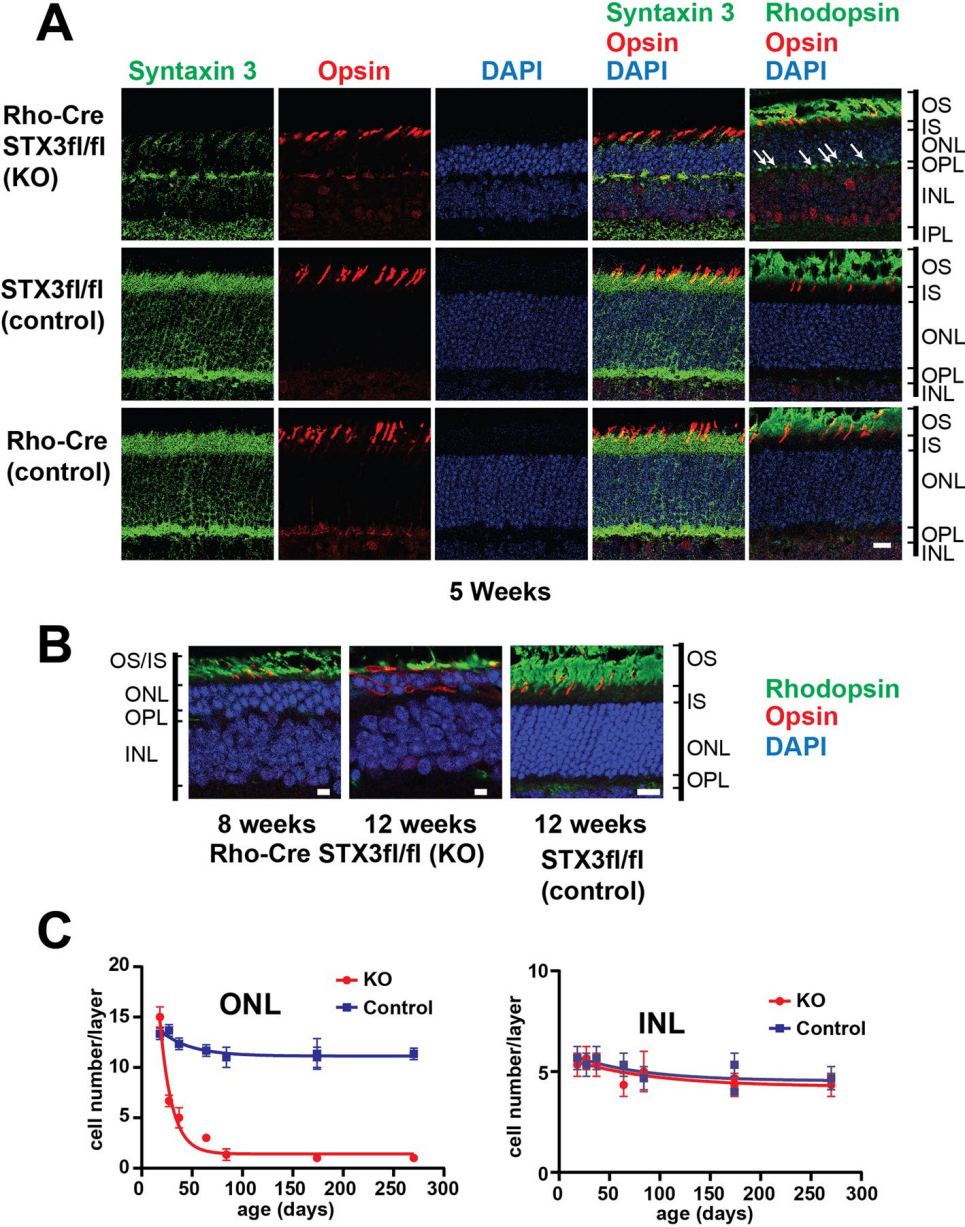
Inactivation of Stx3 in rod photoreceptors leads to progressive degeneration. **a** At 5 weeks of age there is a ≈60% reduction of photoreceptors in the knockout animals compared to littermate controls. Rhodopsin and cone opsin are still found in the outer segments, but aberrant distribution of rhodopsin is observed in the OPL and ONL of the knockout animals (arrows). **b** Analysis of older animals shows dramatic degeneration of photoreceptors after 8 and 12 weeks of age in the KO animals compared to controls. **c** Quantification of retinal layer cell numbers in the KO animals vs. controls over time shows the rapid specific degeneration of photoreceptors within several weeks of inactivation of Stx3 in the rod photoreceptors at day 18. Each data point represents quantification of three randomly selected areas of the retina obtained from one animal with ± SEM. Datapoints were fitted with the exponential plateau least squares fit method using PRISM 8 (Graphpad). The dramatic loss of photoreceptors was observed in six different knockout animals > 4 weeks of age. *IS* Inner segments, *OS* outer segments, *ONL* outer nuclear layer, *OPL* outer plexiform layer, *INL* inner nuclear layer, *IPL* inner plexiform layer, *GCL* ganglion cell layer. Scale bars: 20 μm

At age 5 weeks, a ≈ 60% decrease in the thickness of the outer nuclear layer (ONL) and in the number of neuronal somata in the ONL was observed (Fig. 4a, c) indicating that a large number of photoreceptors had died. This demonstrated that *Stx3* expression is essential for the survival of retinal photoreceptors. At 5 weeks of age, *Stx3* was still expressed in the cones in the rod-specific conditional knockout animals, and the cone outer segments were morphologically grossly normal, with a small reduction in length and displacement of the outer segments and some ectopic localization of opsin to the OPL (Fig. 4a). In some of the remaining rods, rhodopsin was appropriately localized to the outer segments, but a marked amount of rhodopsin mislocalized to the OPL (Fig. 4a, arrows). These findings suggest that while rhodopsin can be trafficked to the ROS in the absence of Stx3, its proper trafficking in the rod is partially affected by the lack of Stx3.

At 8 and 12 weeks of age, an increasing cell loss and an increase in the ectopic expression of rhodopsin were observed. Cone photoreceptor loss and the ectopic expression of opsin in cones was also observed (Fig. 4b, c). The progressive degenerative phenotype was quantified by analyzing the cell numbers in the ONL and INL at different time points (Fig. 4c). This analysis revealed a rapid loss in the number of neuronal somata in the ONL, indicative of photoreceptor death, whereas the number of cells in the INL, wherein the somata of horizontal cells, bipolar cells and amacrine cells reside, was no different from controls.

## Discussion

We report here that individuals with MVID and with biallelic *STX3* loss-of-function variants that affect both *STX3A* and *STX3B* transcript isoforms display visual impairment consistent with an EOSRD. We show that *STX3B* rather than *STX3A* is highly expressed in the human retina, where the protein is found in the inner and outer segments of rod and cone photoreceptors, and in both plexiform layers, where the synaptic endings of photoreceptors and bipolar cells reside. Inactivation of *Stx3* in murine rod photoreceptors resulted in their rapid degeneration as shown here and in a very recent study (Kakakhel et al. 2020). The non-cell autonomous loss of cones in our knockout animals is likely attributable to a bystander effect, as seen in a number of retinal disorders (Brockerhoff and Fadool 2011; Lewis et al. 2010). Collectively, our findings in human subjects and mice establish that STX3 has an essential role in photoreceptor survival and vision.

In addition to visual impairment and MVID, global developmental delay was present in a few subjects with pathogenic *STX3* variants (Chograni et al. 2015; Julia et al. 2019). The delay in patients from our case series is mild and might be largely attributable to the extensive hospitalizations, immobility caused by dependency on parenteral nutrition and severe visual impairment. Our examination of human brain samples did not indicate significant levels of STX3 expression overall, which is consistent with previous studies that have found the lowest level of STX3 expression in brain compared to other tissues in humans and rodents (Bennett et al. 1993; Curtis et al. 2008; Delgrossi et al. 1997). However, we cannot exclude the possibility that STX3 may be expressed in a small, specific subset of human brain cells, where it may impact human development or cognition. Early studies in mice suggested that STX3 may be involved in some types of learning and memory (Jurado et al. 2013), but a subsequent study of the same brain region failed to reveal an effect of STX3 inactivation on basal synaptic function or on a specific type of synaptic plasticity or learning and memory tasks (Shi et al. 2020).

How might loss of STX3 function lead to photoreceptor degeneration? STX3 has been implicated in the trafficking of rhodopsin to the outer segments of photoreceptors (Chuang et al. 2007), and the proper trafficking of rhodopsin to its target compartment is essential for the survival of the photoreceptors. For example, pathogenic variants in the rhodopsin gene or in proteins that catalyze the trafficking of rhodopsin lead to degeneration of photoreceptors in humans and in animal models (Mendes et al. 2005). In addition, STX3 has been implicated in the trafficking of the essential outer segment proteins PRPH2 and ROM1 (Zulliger et al. 2015). However, we did not observe a mislocalization of rhodopsin in rods of P18 knockout mice, an age at which the Stx3 gene is expected to be fully inactivated (Li et al. 2005). At age 5 weeks, however, rhodopsin was present in the outer segments and partly mislocalized to the OPL, indicating that proper rhodopsin trafficking is affected in the absence of Stx3. Similarly, a recent study using conditional knockout mouse lines that had Stx3 inactivated in rods as well as cones during early development, showed that rhodopsin, PRPH2 and ROM1 can get transported to the outer segments in the absence of Stx3 (Kakakhel et al. 2020). However, this study also found a significant amount of mislocalized rhodopsin, PRPH2 and ROM1 in photoreceptors that lack Stx3. The presence of mislocalized rhodopsin, PRPH2 and ROM1 might alter the physical properties, the composition, and the function of the affected compartments in photoreceptors, as suggested in the case of accumulation of truncated rhodopsin in various ectopic membrane locations (Deretic 2006). This pathological process could contribute to photoreceptor cell death by effecting protein degradation and by destabilizing the outer segment and its assembly, and by decreasing the availability of functional proteins important for the phototransduction process in the regions where the aberrant rhodopsin is present (Mendes et al. 2005).

Previously, our study of duodenal biopsies from patient P2 showed a complete loss of STX3 labeling, identified an abnormal subapical vesiculo-tubular network as abnormally extended, aberrant apical recycling endosomes, and indicated that disrupted trafficking between cargo vesicles and the apical plasma membrane is the primary cause of a defect of epithelial polarity and subsequent facultative loss of brush border integrity (Vogel et al. 2017). This membrane recycling defect supposedly leads to the symptoms of MVID, and supports hypotheses of trafficking or membrane recycling defects in photoreceptor cells of persons with STX3 deficiency.

STX3 in photoreceptor and bipolar cell synaptic endings has been suggested to play a critical role in synaptic transmission (Curtis et al. 2010; Datta et al. 2017; Hays et al. 2020). Conceivably, degeneration and death of photoreceptors could result from the disruption of neurotransmitter release from photoreceptors. However, in mouse models that inactivate the retina-specific Ca_*v*_1.4 calcium channel, a protein essential for neurotransmitter release from rod photoreceptors, degeneration of photoreceptors is not observed (Mansergh et al. 2005). Thus, while loss of STX3 function may prevent the transmission of visual information from photoreceptors and bipolar cells to downstream neurons, there is no evidence to suggest that the loss of synaptic transmission itself is lethal to these neurons.

Syntaxins are also important for the trafficking of membrane itself and for membrane repair. STX3, in particular, has been shown to play a critical role in cell membrane expansion and neurite outgrowth (Darios and Davletov 2006). Together with the localization pattern of STX3 in human photoreceptors, this raises the possibility that STX3 may have a more general role in photoreceptor survival and the assembly and renewal of the human photoreceptor outer segment. This hypothesis is supported by studies that deleted or inactivated the plasma membrane syntaxins 1A and 1B from hippocampal (non-ciliated) neurons causing them to die in a manner unrelated to a defect in synaptic transmission (Peng et al. 2013; Vardar et al. 2016) and that the introduction of exogenous STX3 was able to rescue these neurons (Vardar et al. 2016). STX3 is the only plasma membrane-associated syntaxin expressed in photoreceptors (Sherry et al. 2006), and other syntaxins are only expressed at low levels (Shekhar et al. 2016). STX3 might participate in cellular house-keeping functions, and the observed mislocalization of outer segment proteins and synaptic phenotypes might represent, at least in part, an epi-phenomenon of dying photoreceptors.

The visual phenotype in humans that carry homozygous pathogenic *STX3* variants might thus be caused by photoreceptor degeneration resulting from defective trafficking, from synaptic defects, or from disruption of more general cellular functions. Each of these must be considered when designing strategies for vision restoration for these subjects.

More than 200 genes associated with inherited photoreceptor degeneration have been identified in humans (Lee and Garg 2015), and their pathogenic variants cause diseases that can show differences in age of onset, course of the disease and additional (syndromic) organ involvement. Pathogenic variants affecting both the STX3A and STX3B isoforms cause a recognizable type of syndromic retinal disease, characterized by the unique combination of a congenital and intractable diarrhea and EOSRD. Before this study, the existence of the human syntaxin 3B had not been demonstrated, and it was not part of the annotated transcripts encoded by the human STX3 gene. Therefore, mutations affecting the STX3B transcript-specific exons may have been missed in previous large scale genomic screens for mutations implicated in human photoreceptor degeneration disorders. The STX3B-specific exons 9B and 10B code for essential regions of the syntaxin protein. Biallelic mutations in these retina-specific exons would cause a new subtype of non-syndromic photoreceptor degeneration.

## Data and code availability

The sequence of the *STX3B* transcript expressed in the retina that is generated by differential splicing has been deposited to GenBank (Accession number: MW273340). All the other data supporting the findings of this study are available within the article and from the corresponding authors upon request.

## Supplementary Information

Below is the link to the electronic supplementary material.

Supplementary file1 Supplementary Fig. 1. Simplified family trees, segregation and Sanger chromatograms relating to novel STX3 variants (TIFF 3415 KB)
