# Supplementary figures and images for: Pathogenic STX3 variants affecting the retinal and intestinal transcripts cause an early-onset severe retinal dystrophy in microvillus inclusion disease subjects
Source: Hum Genet. 2021 May 11;140(8):1143–56. doi: 10.1007/s00439-021-02284-1 (PMC8263458; doi:10.1007/s00439-021-02284-1)

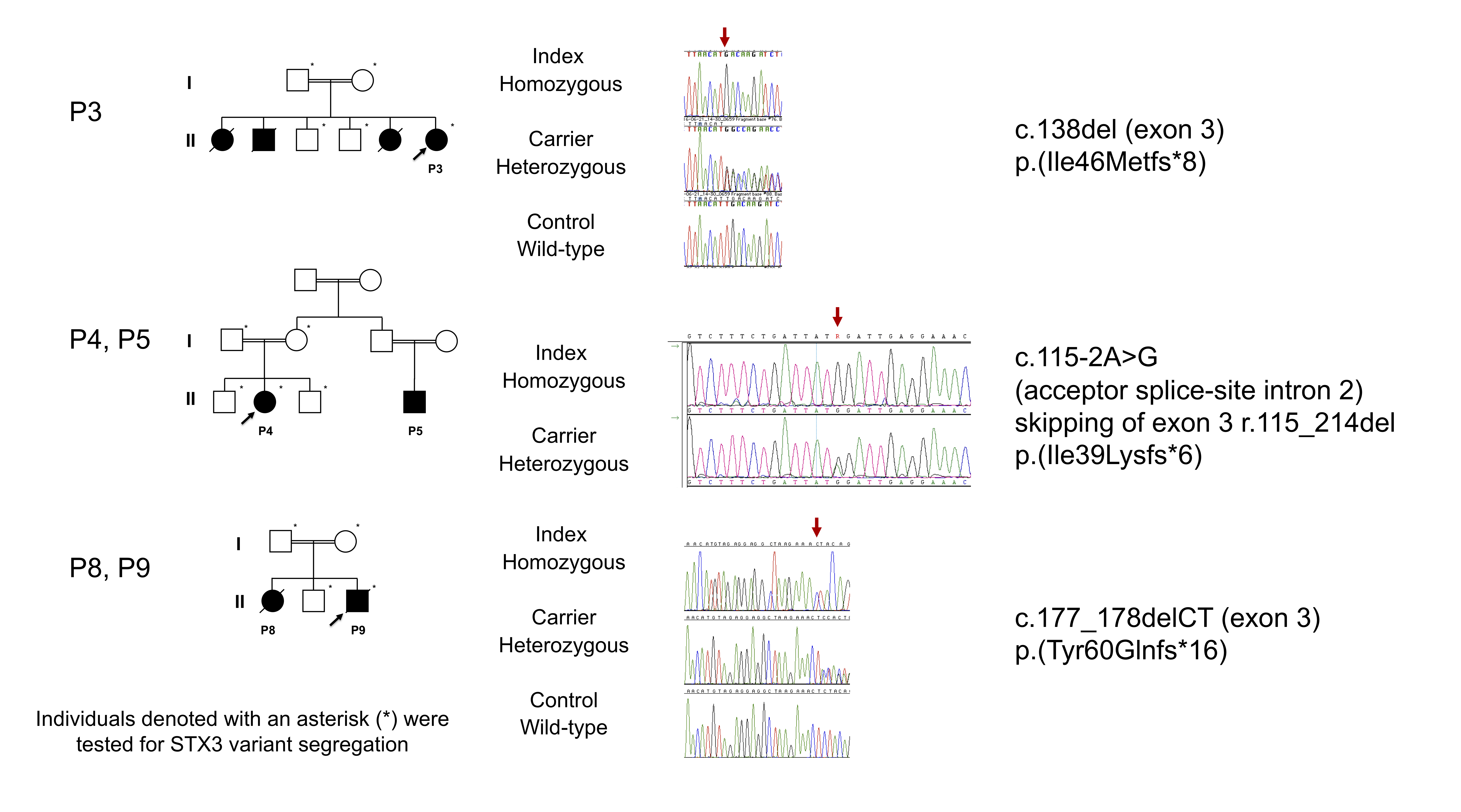

Supplement: Supplementary file 1 — Supplementary file1 Supplementary Fig. 1. Simplified family trees, segregation and Sanger chromatograms relating to novel STX3 variants (TIFF 3415 KB) [file 439_2021_2284_MOESM1_ESM.tiff]
